# The ventral midline thalamus coordinates prefrontal–hippocampal neural synchrony during vicarious trial and error

**DOI:** 10.1038/s41598-022-14707-8

**Published:** 2022-06-29

**Authors:** John J. Stout, Henry L. Hallock, Allison E. George, Suhaas S. Adiraju, Amy L. Griffin

**Affiliations:** 1 Department of Psychological and Brain Sciences, University of Delaware, Newark, DE 19716 USA; 2 Neuroscience Program, Lafayette College, Easton, PA 18042 USA; 3 Lieber Institute for Brain Development, Johns Hopkins Medical Campus, Baltimore, MD 21205 USA

**Keywords:** Cortex, Hippocampus, Spatial memory, Working memory, Decision

## Abstract

When faced with difficult choices, the possible outcomes are considered through a process known as deliberation. In rats, deliberation is thought to be reflected by pause-and-reorienting behaviors, better known as vicarious trial and errors (VTEs). While VTEs are thought to require medial prefrontal cortex (mPFC) and dorsal hippocampal (dHPC) interactions, no empirical evidence has yet demonstrated such a dual requirement. The nucleus reuniens (Re) of the ventral midline thalamus is anatomically connected with both the mPFC and dHPC, is required for HPC-dependent spatial memory tasks, and is critical for mPFC-dHPC neural synchronization. Currently, it is unclear if, or how, the Re is involved in deliberation. Therefore, by examining the role of the Re on VTE behaviors, we can better understand the anatomical and physiological mechanisms supporting deliberation. Here, we examined the impact of Re suppression on VTE behaviors and mPFC-dHPC theta synchrony during asymptotic performance of a HPC-dependent delayed alternation (DA) task. Pharmacological suppression of the Re increased VTE behaviors that occurred with repetitive choice errors. These errors were best characterized as perseverative behaviors, in which some rats repeatedly selected a goal arm that previously yielded no reward. We then examined the impact of Re suppression on mPFC-dHPC theta synchrony during VTEs. We found that during VTEs, Re inactivation was associated with a reduction in mPFC-dHPC theta coherence and mPFC-to-dHPC theta directionality. Our findings suggest that the Re contributes to deliberation by coordinating mPFC-dHPC neural interactions.

## Introduction

When confronted with difficult decisions, the available outcomes are considered through a process known as deliberation. This cognitive process is thought to occur when rats pause and serially look towards potential choices. Collectively, these behaviors are called vicarious trial and errors (VTEs)^1–13^. VTEs emerge when flexible decision-making is required to solve a task^6,9–11,14,15^. For example, Bett et al.^9^ required rats to learn spatial discriminations on a Y-maze. They found that VTEs emerged before rats discovered where a reward was located, but decreased in frequency after the reward location was identified. Similarly, Steiner and Redish^14^ trained rats to perform spatial discriminations on a T-maze, then switched which goal arm contained the reward. They found that VTEs emerged in tandem with these rule-contingency switches, suggesting that VTEs are associated with task manipulations that require rats to adapt their decision making strategies.

Like deliberation in humans, VTEs in rats are thought to rely on hippocampal (HPC) function^12^, an idea supported by studies using behavioral and/or brain manipulations and neural recording approaches. For example, two independent studies reported that VTEs were more prevalent when rats were required to depend on hippocampal-dependent “place” strategies, when compared to striatal-dependent “response” strategies^10,11^. In support of these behavioral results, lesions targeting the HPC^6^, or systemic injections of the NMDA antagonist MK-801 known to disrupt HPC function^16^, lead to significantly fewer VTEs as rats learned discrimination rules. And importantly, in a cornerstone study by Johnson and Redish^8^, spiking activity from HPC neurons was recorded as rats discriminated between spatial choices on multiple paradigms. Using Bayesian decoding, it was discovered that HPC representations swept ahead of the rat at the choice point on a T-maze, alternating between potential choices in a serial manner. These “non-local” spatial coding events were further discovered to occur during VTEs. Thus, mirroring the purported serial process of deliberation^12^, HPC place cells represented choices serially.

While HPC function is intimately tied to VTEs^6,8^, it does not work in isolation. Instead, it is thought the HPC supports deliberation by coordinating its neural computations with regions involved in executive function, like the medial prefrontal cortex (mPFC)^12^. Indeed, the mPFC is critical for both flexible decision making^17–19^, and VTEs^20–22^. In a recent report, Kidder et al.^22^ used optogenetic stimulation to disrupt mPFC function during a spatial delayed alternation (DA) task, a task that probes spatial working memory (SWM). They found that disrupting the mPFC impaired decision making in a manner consistent with reduced VTEs. Thus, like the HPC, the mPFC is critical for VTEs. However, whether these regions communicate in support of deliberation is unknown.

Although there are multiple pathways by which the mPFC and HPC can interact, one of the most prominent projection pathways connecting the HPC with the mPFC is the nucleus reuniens (Re) of the ventral midline thalamus^23–25^. The Re is bi-directionally connected with the mPFC and HPC^26,27^, and sends collateral projections to both regions^27,28^. Consistent with its anatomical connectivity, inactivation of the Re impairs choice accuracy on tasks that require both the mPFC and HPC to perform^29–36^, and supports mPFC-HPC neural interactions^34,37,38^. Thus, the Re nucleus poses as a strong candidate to coordinate mPFC-HPC neural activity during VTEs.

Here, we assessed the impact of Re inactivation on VTEs by combining a previously published dataset from our lab^34^ with newly recorded data. We found that Re inactivation did not cause a general increase in VTEs, but rather caused a marked increase in VTEs occurring on repeated choice errors. We then show that theta coherence, a metric quantifying the strength of the relationship between two LFP signals in the theta (5–10 Hz) range, was reduced under Re suppression, specifically during VTEs. Finally, we used Granger prediction to test whether mPFC-dHPC interactions were disrupted during VTEs in a directionally specific manner. Consistent with disrupted theta coherence, we found reduced mPFC-to-dHPC theta directionality during VTEs. Our findings indicate that the ventral midline thalamic Re nucleus coordinates the deliberative component of VTE by supporting mPFC-dHPC neural interactions.

## Methods

### Subjects

Subjects were 9 (8 male, 1 female) adult Long-Evans hooded rats. Data from 7 males implanted with dual site tetrode recordings from the mPFC/dHPC and a cannula targeting the Re were used from a previously published paper^34^. The remaining 2 rats were implanted with stainless steel wires targeting the mPFC and dHPC and a cannula targeting the Re. All methods were approved and were performed in accordance with guidelines and regulations set by the University of Delaware Institutional Animal Care and Use Committee (IACUC), and are consistent with the ARRIVE guidelines.

### Behavioral apparatus and testing room

**Figure 1:**
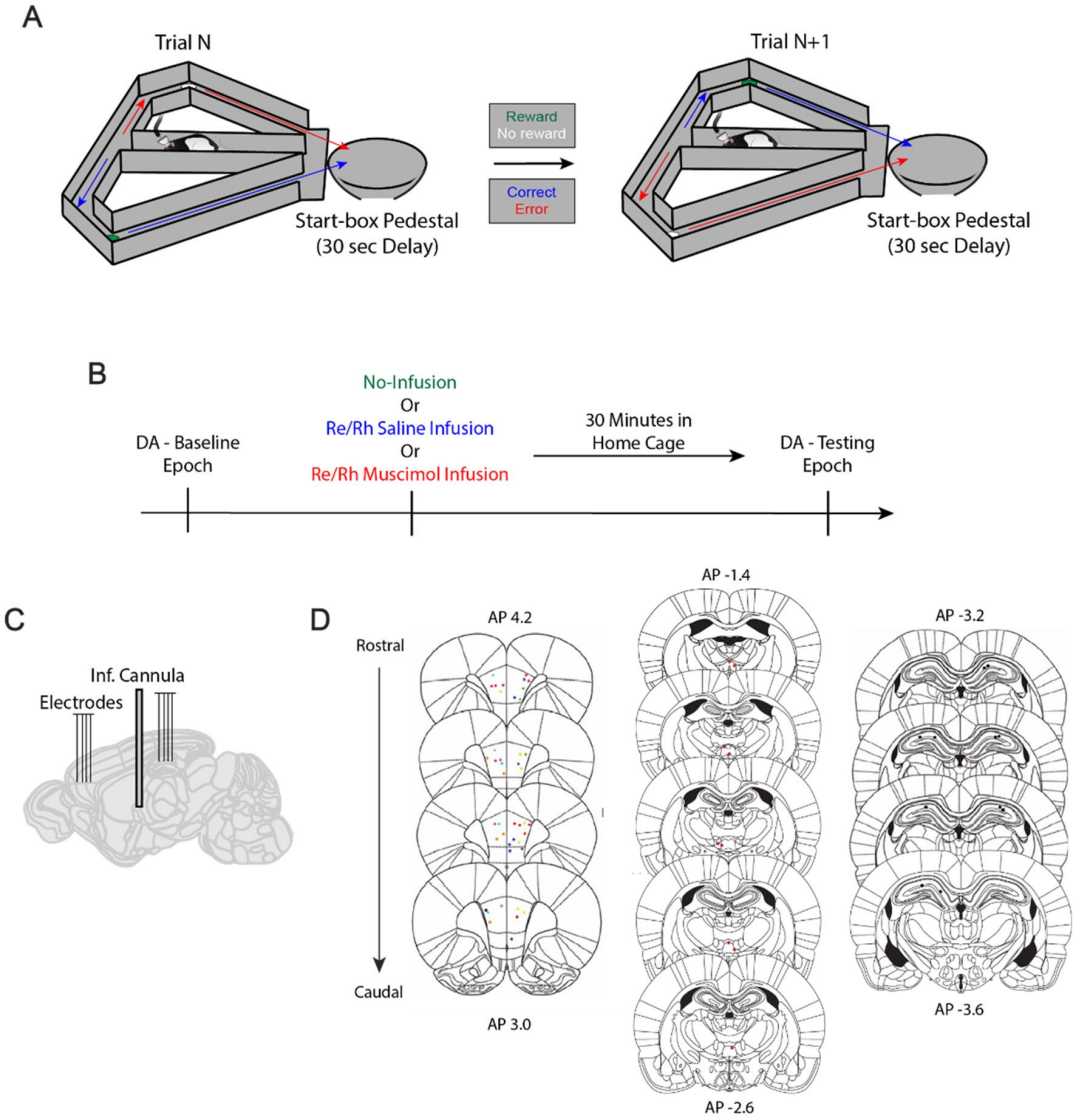
Experimental design. (**A**) Delayed Alternation task schematic. Each trial requires the rat to select the goal arm opposite to the goal arm chosen on the previous trial to receive food reward. All trials, including the first trial of the session, are free-choice trials. A 30 s delay period separates trials, during which the rat is confined to the start-box. Green cup indicates reward, white cup indicates no reward. Blue arrow indicates correct trajectory, red arrow indicates incorrect trajectory. (**B**) During the baseline epoch, rats performed a set of 12–30 trials (DA Baseline epoch). They then received an infusion of saline, infusion of muscimol, or no infusion and returned to their home cage. Thirty minutes later, rats were tested on another set of 12–20 trials (DA Testing Epoch; see Hallock et al.^34^). (**C**) Schematic of recording sites in the mPFC and dHPC and with a cannula site in the Re/Rh. (**D**) Histological confirmations of recording and cannulae placements. Colored dots in the mPFC (left) indicate different tetrode placements (see Hallock et al.^34^).

Behavioral training and testing was carried out on a modified T-maze (Central stem: 116 × 10 cm, goal arms (2): 56.5 × 10 cm each, return arms (2): 112 × 10 cm each). The start box, where rats were confined with a wooden barricade between trials, was located at the base of the maze stem (Fig. 1A). The maze was located in a dimly lit room and surrounded by black curtains with attached visual cues (red and green tape strips, triangles, and patterned circles).

### Handling, pre-training, and task training

Handling and pre-training procedures were similar to previous studies from our lab^32,34^. First, rats were handled for 10–15 min per day for 5 days. They were then shaped to consume chocolate sprinkles located in plastic bottle caps at the goal zone of the T-maze. Rats were given 3 min to consume the reward, and were then placed into the opposite goal zone. Once rats consumed the reward within 90 s on all 6 goal zone visits, they were moved onto the next stage of behavioral shaping, forced runs. Prior to each forced run trial, the experimenter placed a barricade in the entry point of the left or right goal arm (6 left, 6 right trials in a pseudorandom sequence). Each trial began with the rat confined in the start box by a barricade. Once the barricade was removed, rats then traversed the central stem of the maze, turned into the open goal arm, and were rewarded at the goal-zone. After reward consumption, rats returned to the start box via the return arm. Once rats consumed the reward and returned to the start box on 10/12 trials for two consecutive sessions, or 12/12 trials for one session, they began delayed alternation (DA) task training. During DA training, rats were rewarded for alternating visits to the left and right goal arms. Similar to the forced run trials, after choosing a goal arm, the rats returned to the start box via the return arm where they were confined by a wooden barricade for 30 s. All rats included in the study were trained until they reached 80% proficiency on the DA task for two consecutive days (i.e. correct choices on 20/24 trials). Two of the rats that produced data for this report were added after the Hallock et al.^34^ dataset was completed. These rats were initially trained on a separate maze and underwent continuous alternation (CA) training, which was identical to DA training, except that rats did not return to the start box. Consistent with the published dataset, prior to behavioral testing, these rats were shaped to perform the DA task at 80% correct for two consecutive days on a T-maze.

### Surgery

Rats were implanted with a micro-drive loaded with independently moveable tetrodes (7/9 rats), or stainless steel wires targeting both the mPFC and the dHPC (2/9 rats) (Fig. 1C). References were either local (mPFC or HPC; N = 7/9 rats), or in the case of stainless steel wires, were referenced to a cerebellum wire (N = 2/9 rats). Surgical details for microdrive and cannula placement can be found in our previous publication^34^. Two stainless steel wires targeting the mPFC were implanted at an 8-degree angle, 3.1 mm anterior to bregma and 1.0 mm lateral. 4 stainless steel wires with dorsoventral offsets ranging from 0.5 to 1 mm were implanted 3.7 mm posterior and 2.2 mm lateral to bregma. Two cerebellum reference wires were placed 10 to 12 mm posterior to, and 1.5 mm lateral to bregma.

### Recording and infusion procedures

Each rat underwent three recording sessions, separated by at least 24 h, in which one of three infusion conditions were employed: (1) no infusion (2) saline infusion into the Re, and (3) muscimol infusion into the Re (Fig. 1B). For each recording session, each rat performed a baseline epoch in which LFP was recorded from the dHPC and mPFC while the rats performed a session of DA trials. Immediately after the baseline epoch, the rats underwent one of the three infusions (no infusion, saline infusion, or muscimol infusion, followed 30 min later by another recording epoch (“testing”) during which the rat performed another set of DA trials.

Rats were given infusions either of saline (1X PBS, Fisher Scientific) or muscimol, a ${GABA}_{A}$ receptor agonist (Life Technologies Solutions). Muscimol was diluted to a concentration of 0.25 μg/μL in PBS and infused using a microinfusion syringe (Hamilton) and automated infusion pump (World Precision Instruments), at a rate of 0.25 μL/min and total volume of 0.5 μL.

### Perfusion and histology

Rats were given a 0.5 μL volume infusion of fluorophore-conjugated muscimol (BODIPY TMR-X; Life Technologies) 30 min before perfusion^39^. Cannulae placements were visualized via staining half of the Re brain slices with cresyl violet, and the other half with ProLong Gold with DAPI (Life Technologies), highlighting the spread of fluorophore-conjugated muscimol. Cannulae and electrode track verifications (Fig. 1D) were accomplished by superimposing digital plates from the Paxinos and Watson^40^ rat brain atlas over pictures of the cresyl-stained brain slices using Adobe Illustrator. One rat was perfused without the internal cannula, and the Re implant location was estimated based on TMR fluorescence. All rats were confirmed to have a combination of TMR fluorescence and cannula tips in the ventral midline thalamus, primarily targeting the Re (Supplementary Fig. S1). Imaging was performed using an LSM710 Zeiss or Leica Stellaris 8 (supported via NIST 70NANB21H085).

### Identifying VTE behaviors

VTE is defined by pause-and-reorienting behaviors at choice points^12^. While this umbrella term includes the classic head-sweeping behaviors^2^, it also includes instances when rats simply pause at the choice point before making their decision^6^. To ensure we extracted both types of events, we took two independent approaches. First, we used the commonly-employed integrated absolute change in angular velocity (IdPhi) method^12^. The choice point was visually identified for each session as a square space surrounding the divergence in trajectory towards the goal-arms, which included position data that immediately preceded choice-point entry (see Fig. 4A). Position data during choice-point passes were smoothed using a Gaussian-weighted moving average with a window length of 30 samples (reflects the sampling rate of ~ 30 samples/s) using MATLAB’s *smoothdata* function. IdPhi was calculated based on position data obtained via LED tracking of the rats’ head-stage^15^ using modified MATLAB code provided by D. Redish. Briefly, for 2-dimensional position data, velocity in the X (‘dX’) and Y (‘dY’) dimensions were obtained using a discrete time-adaptive windowing method^41^. Phi was then defined by taking the atan2 of dY and dX. Next, the change in movement orientation (dPhi) was estimated by applying the time-adaptive windowing method to the unwrapped phi estimate. The integrated absolute value of change in movement orientation (IdPhi) was defined by taking the integral of |dPhi| estimates. Finally, the natural log and z-score of all IdPhi scores was taken to produce the zlnIdPhi distribution (Fig. S2A). The VTE threshold was estimated by first identifying the two prominent components of the distribution: the “normal” and “tail” components^12^. The “normal” component reflects ballistic-like or non-VTE trajectories which were the predominant trajectory type across trials (see Supplementary Figs. S2B,C). The normal distribution tapered at a zlnIdPhi of 0, which was our data-driven threshold for VTE. In general, these definitions are highly consistent with past work^12,15,42^. Visual inspection of position data was used to confirm this threshold (Supplementary Fig. S2B,C). All putative VTE events were visually inspected using custom-written MATLAB code that ‘played-back’ the rats movements through the choice point. This play-back function was overlaid on position data that demonstrated the rats’ velocity. VTE events were rejected if prolonged pauses occurred well-after the choice point in conjunction with the absence of clear head-sweeping behaviors. Finally, some high-speed head sweeping behaviors resulted in low zlnIdphi scores and were erroneously counted as non-VTE trajectories. To account for this, we developed an approach which extracted head-sweeping VTE events. Simply, we defined a square space surrounding the boundaries of the choice point, then searched for trials where rats entered both left and right goal-arms (Supplementary Fig. S2D). Like above, all candidate VTE head-sweeps were visually inspected (Supplementary Fig. S2E,F) and accepted or rejected on the basis of whether tracking errors were observed, or whether “VTE” behaviors occurred outside of the choice point. Code for both approaches can be found at www.github.com/GriffinLabCode/GriffinCode.

### Spectral analyses

LFP data were extracted during choice-point passes by defining a square space surrounding the T-junction, then organized according to whether they belong to VTE or non-VTE events (Fig. 4A). Next, LFPs were tested for clipping artifacts, large frequency amplitude events that corrupt the signal. Since clipping events are characterized by repeated voltage values, we used custom written MATLAB code that detected multiple voltage repeats (*detect_clipping.m*), then excluded any trial with an LFP signal saturated by 1% of clipping events. Signals were then concatenated into vectors according to whether trials were characterized as VTE or non-VTE. Using LFP from the baseline conditions, power spectra were generated for mPFC and HPC signals using MATLABs *pspectrum*, log transformed, then inspected to define the ‘theta’ range as 5–10 Hz (Fig. 4B). Mean squared coherence was calculated using MATLABs *mscohere* function using a frequency range of 1–20 Hz in 0.5 Hz intervals. No windowing or sample overlap procedures were used to estimate coherence.

Using custom written MATLAB code^34,43^, bivariate Granger prediction was used to assess directionality between the mPFC and dHPC LFP. Specifically, univariate and bivariate autoregressions are used to test whether lagged data in signal “X” can predict future values of signal “Y” better than lagged values of signal “Y” predicts itself. The same is true in the opposite direction (e.g. whether “Y” can predict “X” better than “X” predicts itself). As previously reported^44^, an example univariate model is like so:

$$PFC_{t} = \sum\limits_{{n = 1}}^{k} {a_{n} PFC_{{t - n}} + e_{t} ,}$$

while an example bivariate model is as follows:

$$PFC_{t} = \sum\limits_{{n = 1}}^{k} {a_{n} PFC_{{t - n}} } + \sum\limits_{{n = 1}}^{k} {b_{n} HPC_{{t - n}} + \epsilon _{t} .}$$

For each model, *a* and *b* reflect autoregressive coefficients, *t* reflects the time point for the LFP data, *k* reflects the model order, *n* reflects the lag, *e* represents the variance not explained by a univariate model, while ${\varvec{\epsilon}}$ reflects the variance not explained by the bivariate model. By examining the variance in *e* and ${\varvec{\epsilon}}$, we derive at Granger prediction estimates:

$${GC}_{HPC->PFC}=log\left(\frac{Var[e]}{Var[\epsilon ]}\right).$$

Importantly, these same univariate and bivariate models are applied in the PFC-to-HPC direction, providing two unique Granger prediction estimates for two input signals. To perform frequency-specific Granger prediction, Geweke’s method was performed. Model order was estimated by calculating Bayesian information criteria (BIC) across a range of lags (e.g. 1–20 sampled lags). For each session, the lag providing the smallest BIC value was chosen as a session-dependent model order. Finally, session-dependent model orders were averaged across all sessions, then rounded, to provide one model order to apply across all sessions.

### Statistical analysis

**Figure 2:**
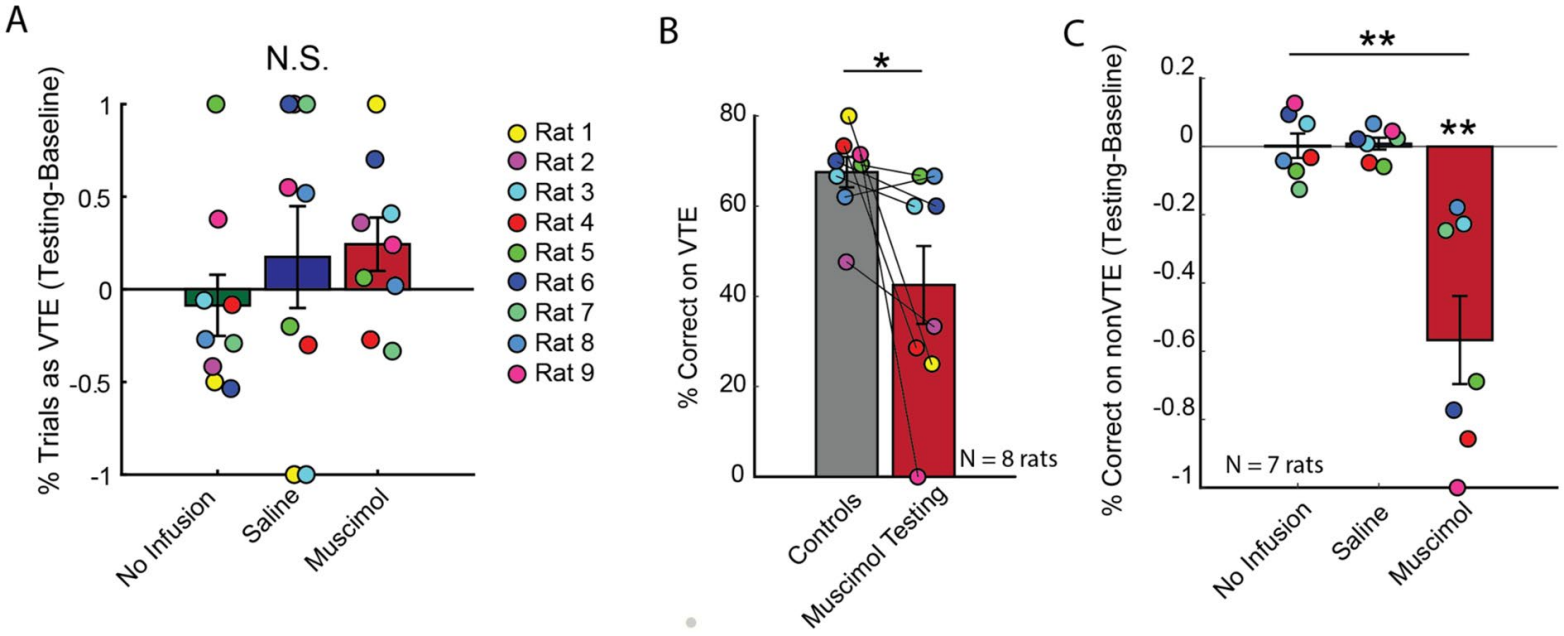
Re inactivation disrupts choice-accuracy during both VTE and non-VTE. (**A**) Re inactivation did not change the percentage of trials with VTE. (**B**) Choice accuracy on trials with VTE, as measured by % correct, was significantly different between control and muscimol groups. Paired t-test with a significance level of 0.05. “Control” refers to collapsing across all sessions except muscimol testing (Supplementary Fig. S3). (**C**) Re inactivation disrupted choice accuracy during non-VTE trials as measured via repeated measures ANOVA, then with t-tests against a null of 0 (significance threshold is 0.0167). *p < 0.05. **p < 0.01. ***p < 0.001. Data are displayed as the mean ± s.e.m.

**Figure 3:**
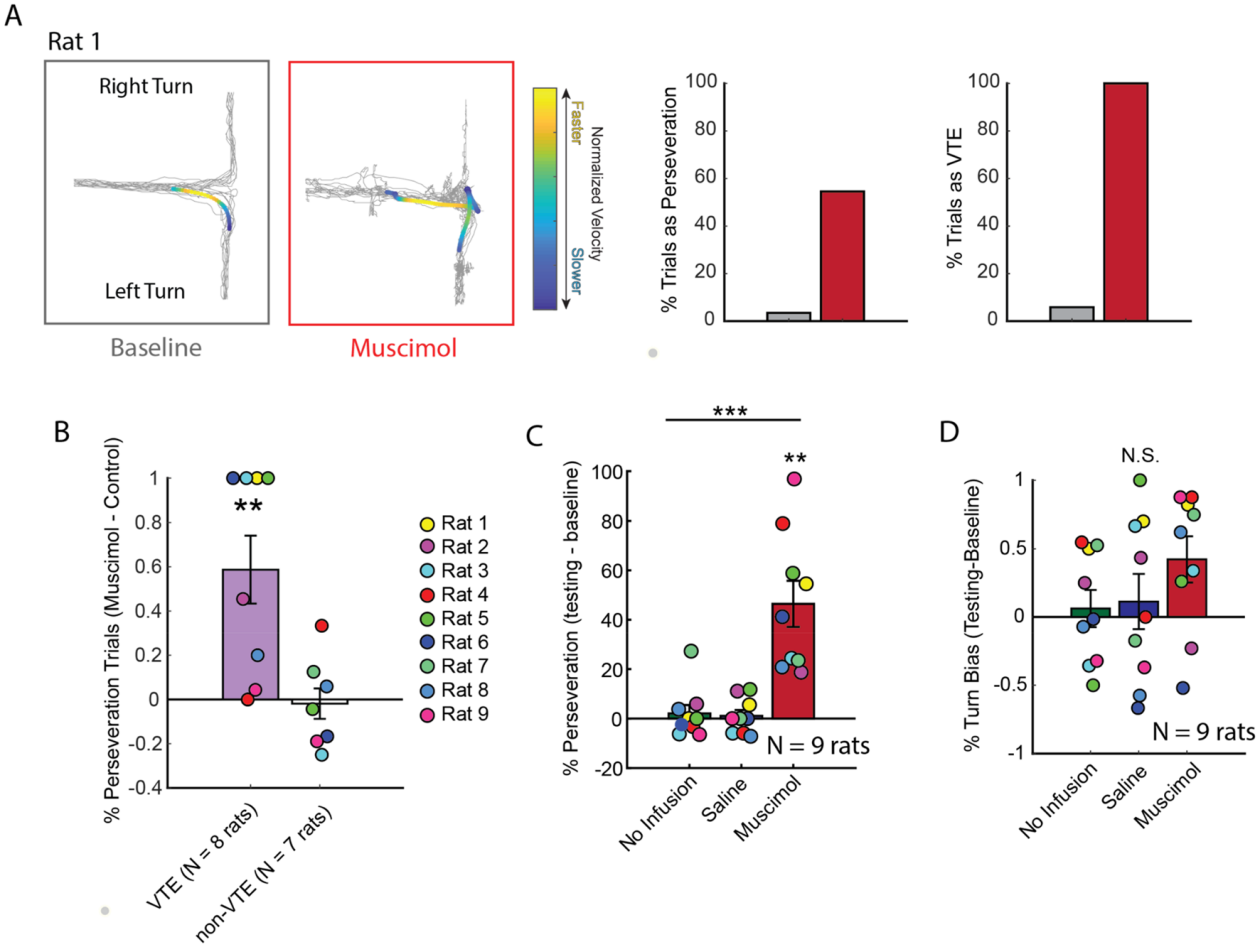
Re inactivation increased VTEs on choice error sequences. (**A**) An example rat that exhibited VTEs on every trial following Re inactivation, with > 50% of trials being perseverations. Left panels display position data from an example baseline (gray) and muscimol testing (red) trial. Overlaid are example trajectories where warm colors indicate greater normalized head velocity. Bar graphs (right) demonstrate that this example rat exhibited both perseverative and deliberative choice behaviors during muscimol testing (red) which were less prevalent across the various control conditions (gray) (see Rat 1 Fig. 2A). (**B**) Percentage of perseveration trials that were VTEs or non-VTEs were used to generate a normalized difference score between control and muscimol sessions. Notice that Re inactivation caused a significant increase to perseveration trials with VTEs but not to perseveration trials with non-VTEs. (**C**) In general, % perseveration was increased by Re inactivation. (**D**) Unlike perseveration, which makes no assumption of spatial bias, turn bias reflects a difference between the amount of left and right choices (e.g. |#left–#right|/#trials). Re inactivation did not lead to a reliable increase in turn-bias as measured via one-way repeated measures ANOVA. Notice that the numerical change in turn bias is best explained by the data in (**C**). Data are displayed as the mean ± s.e.m. **p < 0.01 ***p < 0.001.

One-way repeated measures Analysis of Variance (ANOVA) was used whenever appropriate. Since ANOVA was performed on normalized difference scores, significant effects were followed up with t-tests between baseline and testing conditions and were only considered significant if p < 0.0167 based on Bonferroni’s alpha correction. Each figure panel was considered a separate analysis and was treated as such with alpha corrections. The raw p-values are reported in this manuscript, but significance markers “*” were only placed when significance exceeded Bonferroni alpha levels. “Bonferroni p” reflects a converted p-value, whereby the raw p-value was multiplied by the number of comparisons (e.g. p = 0.05 × 3 = 0.15). Normalized difference scores were estimated for each rat, ${\varvec{i}}$, as such:

$$Norm.\,Diff\,Score_{i} = \frac{{Y_{i} - X_{i} }}{{Y_{i} + X_{i} }},$$

where “Y” and “X” refer to variables in comparison, which were either testing vs baseline for the variety of conditions (see Fig. 2A) or muscimol testing—controls (see Fig. 3B), where “controls” refers to a variable obtained by collapsing across all control sessions (e.g. all sessions except the muscimol testing session). Confidence intervals and Cohen’s D effect sizes (derived by using *computeCohen_d.m* written by Ruggero G. Bettinardi and retrieved from MathWorks) were reported whenever a significant effect was observed. Statistics were performed in RStudio and MATLAB. MATLAB and Adobe illustrator were used to generate figures.

## Results

### Effect of Re inactivation on VTE

Because inactiving the mPFC or HPC was shown to reduce the frequency of VTEs^6,20–22^, we predicted that Re suppression would result in a similar reduction in these deliberative behaviors. However, against our prediction, a one-way repeated measures ANOVA revealed no significant differences in VTE frequency across the no infusion, saline, and muscimol conditions (Fig. 2A; F(2,16) = 0.45, p = 0.64). Next, because Re inactivation leads to clear choice accuracy impairments on the DA task^33,34^, we wondered if this SWM impairment was specific to VTE or non-VTE trials. Since VTE trials were sparse across the various control sessions (e.g. every baseline and testing session except muscimol testing), we collapsed across these control sessions to generate a score that reflected choice accuracy during VTE trials. We noticed that rat #7 contributed 1 VTE trial during the muscimol testing condition and therefore excluded this rat from all VTE-specific analyses (see Supplementary Fig. S3B). Also, on the muscimol testing session, every trial from rat #1 and #2 was a classified VTE event, thus preventing us from using these rats in the non-VTE analyses (Supplementary Fig. S3C). Therefore, our VTE dataset included 8 rats, while our non-VTE dataset included 7 rats. We found that choice accuracy on VTEs was significantly reduced following Re suppression (Fig. 2B; t(7) = 2.5, p = 0.04; ci = 1.6–48.5, d = 0.89; paired t-test; significance threshold = 0.05). With respect to non-VTE trials, choice accuracy was significantly different across conditions (Fig. 2C; F(2,12) = 16.9, p < 0.001; p = 0.006 Greenhouse–Geisser sphericity correction; one-way repeated measures ANOVA), which was explained by choice accuracy reductions between testing and baseline conditions following Re inactivation (No Infusion: t(6) = 0.06, p = 0.95; Saline: t(6) = 0.47, p = 0.65; Muscimol: t(6) = -4.4, p = 0.005, ci = − 0.88 to − 0.25, d = 1.98; t-test against a null of 0; significance threshold = 0.0167). These findings suggest that Re inactivation disrupts choice accuracy on both VTE and non-VTE trials.

### Re inactivation increases VTE on choice error sequences

Along with impaired SWM, Re inactivation was recently shown to cause inflexible decision-making patterns^45^. Specifically, Re suppression caused the repeated selection of a previously incorrect choice, known as perseveration. When visualizing position data, we observed instances where rats would repeatedly select the same erroneous choice, even while exhibiting VTE (Fig. 3A). This observation seemed paradoxical as deliberation should reflect flexible decision-making, while the repeated selection of a previously incorrect choice is an inflexible behavior. We reasoned that, should Re inactivation cause VTEs to occur more robustly with repeated errors, and if the same were not true of non-VTE, then it would suggest that the Re contributes to the *function* of VTE, which is thought to be deliberation^12^.

In order to test this idea, we needed to simultaneously identify perseveration patterns, along with VTE and non-VTE behaviors. Therefore, we next defined perseveration as the repeated (3×) selection of the same goal-arm (e.g. L, L, L is one count of perseveration, or two consecutive choice errors). Within a perseverative trajectory sequence, we knew that the first choice error would lead to a future error (e.g. L → **L** → L). Therefore, we extracted VTE and non-VTE events around the first error in the perseverative sequence, then pooled the data across the control conditions. We then identified the % of VTE that occurred during these error sequences by normalizing the total number of VTE + perseverative events by the total number of perseverative events. The same procedure was done for non-VTEs. Finally, we used normalized difference scores (see “Methods”) to account for between-subject variability. We found that Re inactivation led to significantly more VTEs that occurred with repeated choice errors (t(7) = 3.6, p = 0.008, ci = 0.20–0.97, d = − 0.76, t-test against a null of 0), but the same was not true of non-VTEs (t(6) = − 0.24, p = 0.81, t-test against a null of 0) (Fig. 3B). There was no significant difference observed between the normalized difference scores, although a paired t-test could only be performed on rats with both VTE and non-VTE data (t(5) = 2.18, p = 0.08, Bonferroni p = 0.24, paired t-test). It is important to mention that on average, the majority of perseverations were non-VTE (controls: 76%, muscimol: 74%).

We then ensured that, like previous reports, perseverative errors were the primary behavioral deficit^45^. For this analysis, we did not dissociate VTE and non-VTE trials. By computing non-normalized difference scores (some control sessions had 0 instances of perseveration), we found a significant difference across the no infusion, saline, and muscimol conditions (Fig. 3C; F(2,16) = 16.6, p < 0.001; p = 0.002, Greenhouse–Geisser sphericity correction; one-way repeated measures ANOVA). Follow up t-tests between testing and baseline sessions confirmed this finding, finding a drastic increase in perseverations under Re suppression (No Infusion: t(8) = 0.6, p = 0.57; Saline: t(8) = 0.74, p = 0.48; Muscimol: t(8) = 5.0, p = 0.001, ci = 24.2–65.6, d = − 1.66; t-tests against a null of 0, significance threshold = 0.0167). To contrast this effect against a different, albeit similar choice strategy, we then calculated a turn bias index. This was measured by identifying the |#left–#right|/#trials, and is informative as to whether rats just simply chose one goal arm for the entirety of a session. It is important to note that these metrics are different. If a rat were to perform 10 repeated left turns, then 10 repeated right turns, there would be a turn bias score of 0%, but a perseverative score of 89%. While there were two rats that exhibited clear turn bias, we found no significant differences across conditions (Fig. 3D; F(2,16) = 1.3, p = 0.29; one-way repeated measures ANOVA). It should be noted that the numerical increase in turn bias during the muscimol testing session likely reflects the perseverative findings in Fig. 3C.

These data indicate that (1) Re inactivation leads to a stereotyped behavior—perseveration and that (2) Re suppression led to an increase in VTEs during repeated choice errors.

### Re inactivation reduces mPFC-HPC theta synchrony during VTE

mPFC-dHPC theta coherence, a measure that reflects phase synchrony between the two regions’ LFP, is positively associated with choice accuracy on SWM paradigms^34,46,47^ and is reduced in magnitude by Re inactivation^34^. While theta coherence is positively associated with learning, VTE frequency is negatively associated with learning, such that VTEs emerge in highest frequency when rats are learning task parameters^6,14,16^. However, whether there is a link between mPFC-dHPC theta coherence and VTEs is unclear. Therefore, we next examined the effect of Re suppression on mPFC-dHPC theta coherence during VTE and non-VTE behaviors.

**Figure 4:**
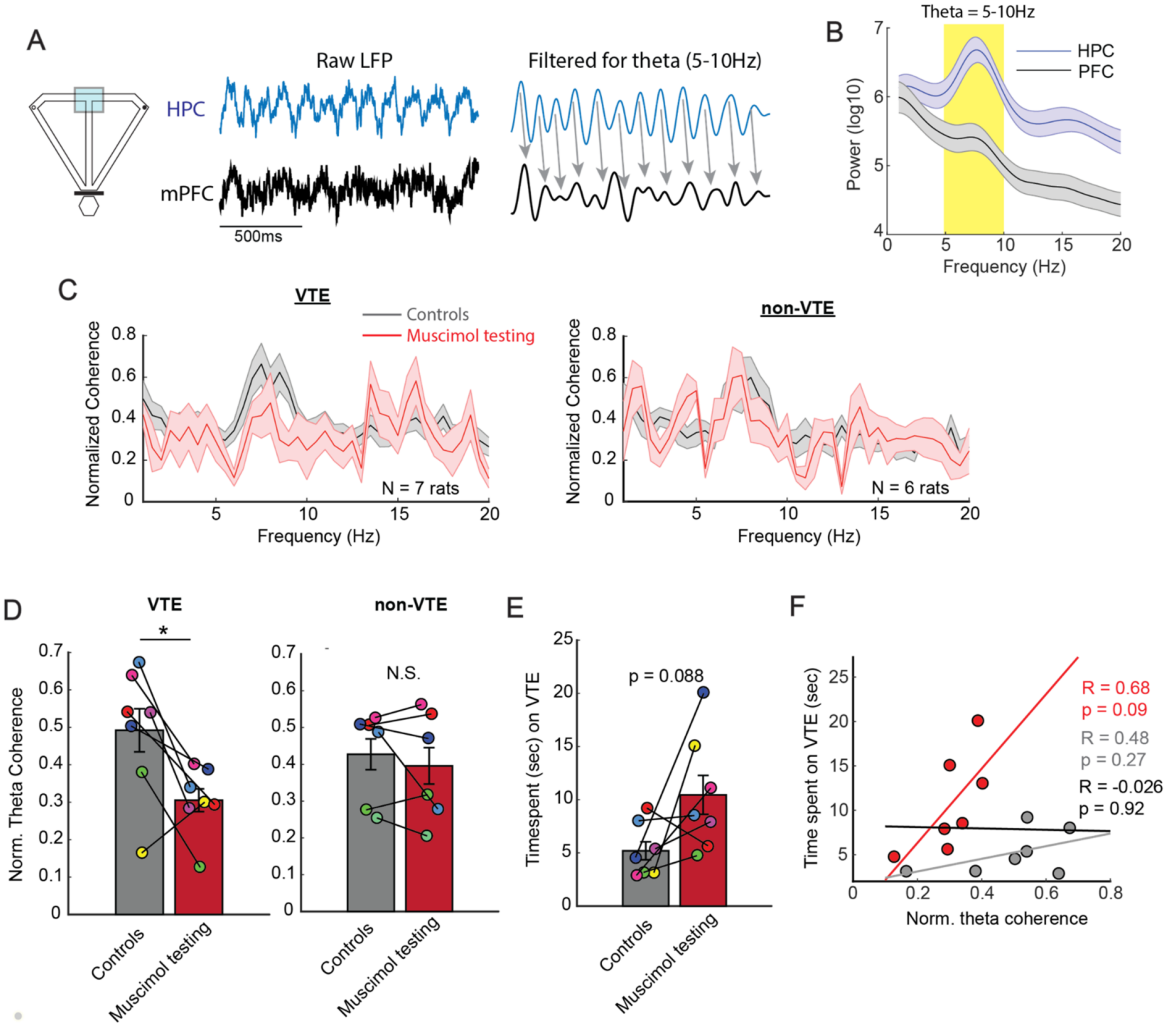
Re inactivation disrupts mPFC-dHPC theta synchrony specifically during VTEs. (**A**) Maze schematic demonstrating that LFP data was extracted from the choice point (blue box surrounding T-junction). Middle panel demonstrates raw LFP. Right panel shows filtered LFP as a conceptual demonstration of theta phase coherence. (**B**) “Theta” was defined as a 5–10 Hz oscillation based on the power spectra from mPFC and HPC LFP. (**C**) Frequency × Coherence plots demonstrating a clear reduction in theta coherence on VTEs (left panel) but not non-VTEs (right panel) after Re inactivation. Red colors indicate data from the muscimol testing session, while gray colors indicate data obtained across control sessions. (**D**) Normalized theta coherence was averaged across the 5–10 Hz range, then statistically compared between control sessions and the muscimol testing session (N = rats). There was a significant reduction in mPFC-dHPC theta coherence during VTE (left panel) but not non-VTE (right panel) trials (paired t-tests, significance threshold of 0.025 for two tests). (**E**) There was no significant difference in time spent at the choice point during VTEs, although the p-value was “trending”. (**F**) Pearson’s correlation was performed between time spent at the choice point and mPFC-dHPC theta coherence. Three separate analyses were performed to isolate control (gray) and muscimol testing (red) datasets (N = 7 rats per group), and then to combine these data (black). Regression lines are color coordinated accordingly. If reductions in mPFC-dHPC theta coherence (**D**) were being driven by increased time spent at the choice point (**E**), then we should observe negative correlations between time spent and theta coherence. Bar graphs and shaded error bars are represented as the mean ± s.e.m. *p < 0.025.

After organizing and processing neural data, we were left with 7 rats with VTE data and 6 rats with non-VTE data. Rat #3 was excluded from all analyses due to mPFC and HPC signals having different sampling rates. Moreover, the rats excluded differed between the grouped data (see Supplementary Fig. S3). To then account for between-subject variability, coherence estimates were first averaged across trials, normalized across frequencies (1–20 Hz), then averaged across rats. The normalization procedure accounts for between subject signal variability as features of oscillations, such as theta, change with recording depth in the hippocampus^48^. LFP was extracted from the choice point (Fig. 4A) and theta was defined as a 5–10 Hz oscillation based on the power spectra (Fig. 4B). To simplify data interpretation, we collapsed across controls for both VTE and non-VTE analyses, and did not perform normalized difference scores as these metrics were already normalized. We found that Re inactivation reduced mPFC-dHPC theta coherence during VTE behaviors (t(6) = 3.17, p = 0.019, ci = 0.04–0.33, d = 1.2), but not during non-VTE behaviors (t(5) = 0.8, p = 0.46; paired t-test; significance level = 0.025) (Fig. 4C,D). Because LFP were extracted around spatially-precise boundaries (e.g. the choice point), it was possible that these effects were being driven by differences in time spent at the choice point. Similarly, since VTEs were less abundant than non-VTEs (Supplementary Fig. S3), it was possible that our results were driven by differences in the amount of data being analyzed. To address both of these concerns, we next focused our analyses on time spent at the choice point during VTEs. We found no significant difference in time spent between muscimol and control groups (Fig. 4E; t(6) = − 2.03, p = 0.088). However, this p-value bordered on what could be considered a “trending” effect. Therefore, we next performed Pearson correlation analyses between time spent at choice point during VTE and mPFC-dHPC theta coherence during VTE. Consistent with our analyses above, we used control and muscimol testing data (N = 7 rats per group). We performed 3 separate correlations between normalized theta coherence and time spent, during VTEs. If increased time spent was driving a reduction in mPFC-dHPC theta coherence, we would expect to find a negative correlation between these variables. First, when collapsing all data, there was no significant correlation between time spent and mPFC-dHPC theta coherence (R = − 0.026, p = 0.92). Second, when we focused on muscimol testing data, there was a strong, albeit non-significant positive correlation between theta coherence and time spent (R = 0.68, p = 0.09). This positive relationship is likely a product of sampling bias as greater time spent should be associated with reduced mPFC-dHPC theta coherence if it were the driving factor in our results (Fig. 4D,E). And third, we found no significant correlation between time spent and theta coherence within the control dataset (R = 0.48, p = 0.27). When visualizing the time-spent data via histogram, we noticed a skew in the central tendency towards low time-spent values. However, log transforming the time-spent variable yielded nearly identical results (All data: R = − 0.0007, p = 0.998; Muscimol: R = 0.76, p = 0.0468; Controls: R = 0.497, p = 0.26). In effect, it is unlikely that our results are driven by (1) differences in gross behavior at the choice point or (2) the sheer amount of data being analyzed from the choice point. We then examined whether Re inactivation disrupted theta power in the mPFC or dHPC. Surprisingly, on VTE trials, Re suppression increased theta power in the mPFC during VTEs (t(6) = -4.0, p = 0.007, ci = − 0.63 to − 0.15, d = − 0.61, paired t-test, significance threshold is 0.025; Supplementary Fig. S4A), but did not change dHPC theta power (t(6) = 1.24, p = 0.26; Supplementary Fig. S4B). On non-VTE trials, Re inactivation did not change mPFC theta power (t(5) = − 1.16, p = 0.3; Supplementary Fig. S4C) nor dHPC theta power (t(5) = 0.46, p = 0.66; Supplementary Fig. S4D). Thus, we show that Re inactivation disrupts mPFC-dHPC theta interactions specifically on VTEs, suggesting reduced mPFC-dHPC functional connectivity during deliberative behaviors.

### Re inactivation disrupts mPFC-to-dHPC theta directionality during VTEs

**Figure 5:**
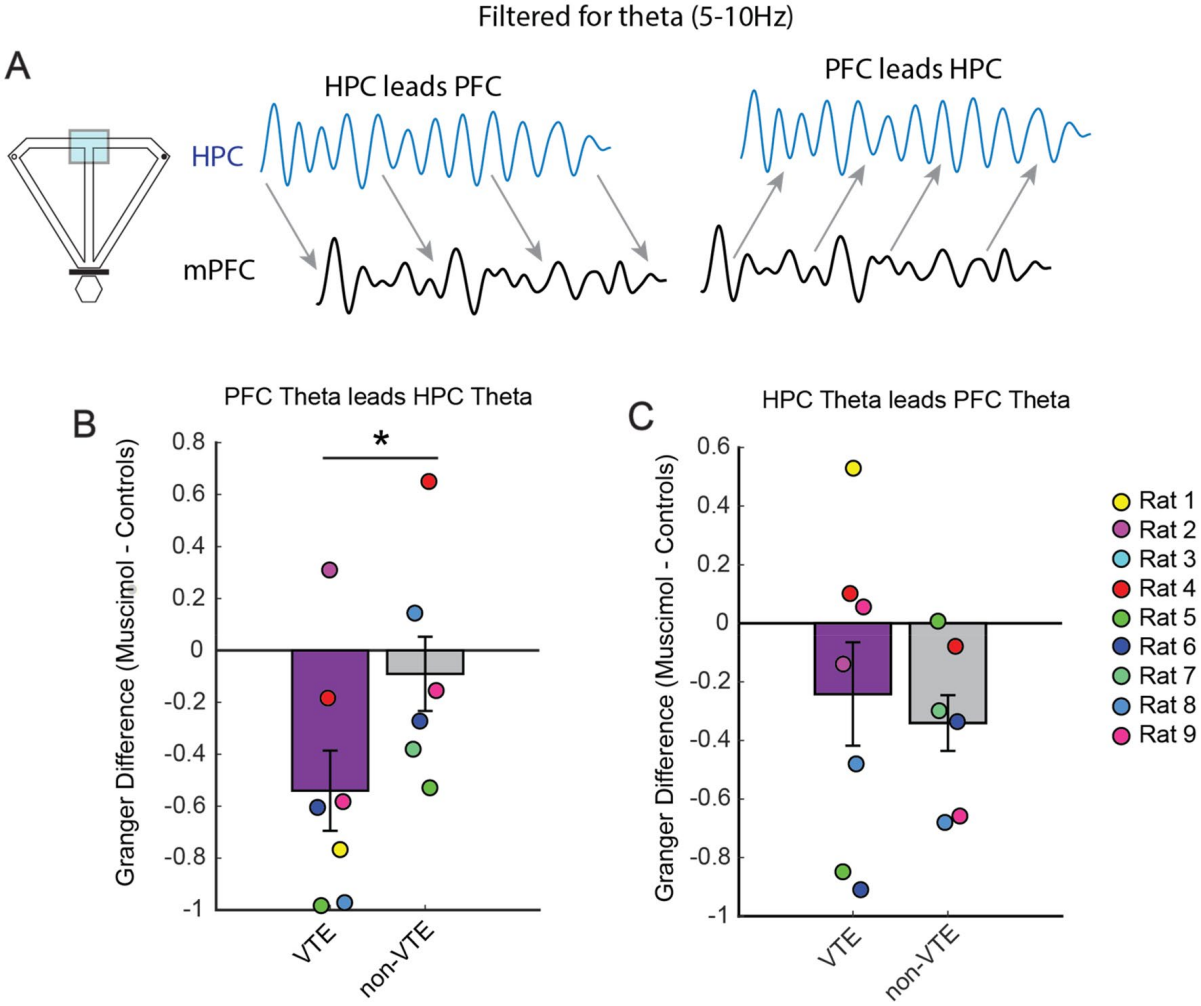
Re inactivation reduces mPFC-to-dHPC theta directionality during VTEs. (**A**) Schematic representing LFP extracted from the choice point (left) and a conceptual representation of Granger prediction (right panels). Notice that Granger prediction provides estimates of predictive power in both directions (e.g. PFC-to-HPC and HPC-to-PFC). (**B**) Re suppression reduced mPFC-to-dHPC theta directionality during VTEs (N = 7) when compared to non-VTEs (N = 6). Note that 5 rats exhibited both VTEs and non-VTEs after reaching inclusion criteria for LFP analyses (see “Results” and “Methods”). (**C**) Re suppression did not disrupt dHPC theta leading mPFC theta. *p < 0.0167. Data are displayed as the mean ± the s.e.m.

The findings thus far indicate that Re inactivation led to increased deliberative behaviors during repetitive error sequences, accompanied by a reduction in mPFC-dHPC theta coherence. To further test how Re suppression disrupted mPFC-dHPC neural interactions during VTE, we turned to bivariate Granger prediction, a statistical approach that approximates directionality within a system. Since bivariate Granger prediction provides estimates of predictive power in two directions (e.g. PFC leads HPC and HPC leads PFC), we focused on each prediction direction separately. Like the results presented in Fig. 4, we focused on LFP extracted from the choice point and extracted Granger causal estimates in the 5–10 Hz range (Fig. 5A). We then averaged Granger causal estimates across the control conditions to compare against the muscimol testing session. While the VTE dataset was made up of 7 rats and the non-VTE dataset was made up of 6 rats, only 5 rats had both VTEs and non-VTEs after meeting inclusion criteria for LFP analyses. Using the Granger prediction estimates, we generated a normalized difference score between the muscimol and control datasets (muscimol − control/muscimol + control). We found that Re inactivation reduced mPFC-to-dHPC theta directionality during VTEs when compared to non-VTEs (Fig. 5B; t(4) = − 4.28, p = 0.013, ci = − 1.0 to − 0.22, d = − 1.36; paired t-test, significance threshold = 0.0167). When we compared muscimol testing to the control dataset, we found a trending reduction in mPFC-to-dHPC theta direction (t(6) = − 3.1, p = 0.022, Bonferroni p = 0.066, ci = − 0.97 to − 0.11, d = − 0.6, t-test against a null of 0, significance threshold = 0.0167), that did not exist during non-VTEs (t(5) = − 0.52, p = 0.63; ttest against a null of 0). Interestingly, the same was not true of dHPC-to-mPFC theta directionality (Fig. 5C). While we did find a trending reduction in dHPC-to-mPFC theta directionality during non-VTEs under Re suppression (t(5) = − 2.9, p = 0.033, Bonferroni p = 0.099; t-test against a null of 0), the same was not true during VTEs (t(6) = − 1.2, p = 0.27; t-test against a null of 0), nor when we compared Granger difference scores between VTE and non-VTEs (t(4) = 0.24, p = 0.83; paired t-test).

Taken together, our findings show that Re inactivation increases deliberation during repeated choice errors, while leading to reduced mPFC-dHPC theta coherence during VTEs that is accompanied by a specific reduction in mPFC-to-dHPC theta directionality. Thus, these findings suggest that the Re nucleus coordinates the communication between the mPFC and dHPC that is associated with deliberation.

## Discussion

In this report, we examined the impact of Re inactivation on decision making behaviors. We predicted that an intact ventral midline thalamus would be important for the manifestation of VTEs, behaviors tied to deliberation^12^. Instead, we found that Re inactivation increased deliberative behaviors occurring within the context of repeated choice errors. These repetitive choice failures, called perseverations, were robust following Re suppression, supporting a previous report demonstrating that Re inactivation leads to inflexible decision making patterns^45^. Analysis of LFP collected from both the mPFC and dHPC showed that Re inactivation reduced mPFC-dHPC theta coherence and reduced mPFC-to-dHPC theta directionality during VTEs. Collectively, our results suggest that the Re nuclei coordinates mPFC-dHPC neural interactions during VTEs, and provides (1) the first report on how the Re nuclei is related to deliberation and (2) the first experiment to identify how disconnecting mPFC and dHPC impacts deliberation.

During the learning of T-maze paradigms with difficult choices, it has been hypothesized that rodents undergo three stages of learning; deliberation, planning, and automation^12^. During the deliberation stage, VTEs exist at high-cost choice-points^49^, and decrease with mPFC or dHPC inactivation^6,9,21,22^. By inactivating the Re, we experimentally disconnected the mPFC from the dHPC, which increased the incidence of flexible decision making behaviors (e.g. VTEs) during inflexible choice patterns (e.g. repeated choice errors). This increase in flexible choice behaviors, despite repeated choice errors, suggests that the Re nucleus may coordinate information for successful deliberation, such as the communication of potential outcomes computed by the HPC^8^ or goal-relevant information from the mPFC^37,50–52^. In support of this idea, mPFC-dHPC theta coherence, a measure of neural synchronization, was reduced during VTEs under Re suppression. The finding that Re suppression did not reduce mPFC-dHPC theta coherence during non-VTE trials may also support this idea, as rats were likely making their choice earlier in the stem (e.g. engaging in route “planning”). Indeed, work from our lab and others has reported that mPFC neurons and their associated neuronal populations are strongly predictive of future choices on SWM paradigms before those choices are made^37,50,52,53^. Thus, we propose that Re suppression did not affect theta synchrony during non-VTE choice trials as rats made their decision earlier in the stem (even if that choice was incorrect). Interestingly, our finding that Re inactivation disrupted mPFC-to-dHPC theta directionality during VTEs is consistent with the hypothesis that during deliberation, the mPFC initiates dHPC sequences in a manner that provides the animal with potential future outcomes^12^. Similarly, the reduced mPFC-to-dHPC theta directionality is consistent with a report by Ito et al.^37^, whereby they suggested that the Re nucleus orchestrates a prefrontal-to-hippocampal directional flow of information. Future studies should test whether Re inactivation disrupts mPFC and dHPC neuronal representations during deliberation. One exciting avenue would be to identify “neuron-pairs” between the mPFC and dHPC^54^, neurons that fire within a short temporal window, and to then identify whether neuronal spike correlations during VTEs are disrupted under Re suppression.

In a previous report^45^, the Re nucleus was pharmacologically suppressed as rats performed a SWM delayed non-match to position (DNMP) task. On this variant of the DNMP task, rats were first required to turn in one direction at the choice point. After a delay period, they were provided a free choice, in which they were rewarded for alternating from their previous trajectory, as in the DA task. However, following an incorrect choice, rats were provided additional opportunities to make a correct decision. Muscimol infusions into the Re nucleus resulted in rats performing error sequences, even when they were given the opportunity to correct an error. Similar to their finding of a “win-shift” failure, we observed that Re suppression led to drastic increases in repetitive choice error sequences. Uniquely, we discovered that Re inactivation increased VTEs on errors that would lead to a future error (e.g. L → **L(VTE)** → L). The finding that VTEs overlapped with error sequences that reflect behavioral inflexibility^45^ suggests that rats were attempting to deliberate, but that this deliberative process failed, likely due to impaired mPFC-dHPC neural interactions. Yet, while our findings suggest that the Re contributes to the deliberative component of VTEs, it is also possible that Re suppression simply disrupted SWM encoding^35^, which would naturally disrupt the deliberative process. Alternatively, it is possible that Re suppression disconnected executive functioning networks with spatial memory networks, causing other competing brain systems to dominate during task performance. For example, Packard et al.^55^ found that fornix lesions facilitated the learning of a striatal dependent task, suggesting that competition exists between the hippocampus and striatum in the intact brain. Future studies are required to determine the specific contribution of the Re nucleus on VTEs. Specifically, the use of rule-switching tasks that induce VTEs, in tandem with Re inactivation procedures and neural recordings, will be necessary to unravel the exact contributions of the Re on deliberation.

In conclusion, we examined the impact of Re suppression on VTE behaviors to answer the question of whether mPFC-HPC communication supports deliberation in rats. By inactivating the Re nucleus, a ventral midline thalamic region that connects the mPFC and HPC, we show that rats attempted to deliberate, but that these deliberations were associated with repeated choice errors. These deliberations coincided with reduced mPFC-dHPC theta coherence, and reduced mPFC-to-dHPC theta directionality, suggesting a disruption in neural coordination. This study provides crucial insight on the contribution of the prefrontal-thalamo-hippocampal circuit to deliberation.

## Supplementary Information

Supplementary Figures.

## Data Availability

Data can be made available upon reasonable request. All code is available on the labs’ github page.
