# Supplementary material for: The ventral midline thalamus coordinates prefrontal–hippocampal neural synchrony during vicarious trial and error
Source: Sci Rep. 2022 Jun 29;12:10940. doi: 10.1038/s41598-022-14707-8 (PMC9243057; doi:10.1038/s41598-022-14707-8)
Supplement: Supplementary file 1 — Supplementary Figures. [file 41598_2022_14707_MOESM1_ESM.docx]

Supplemental


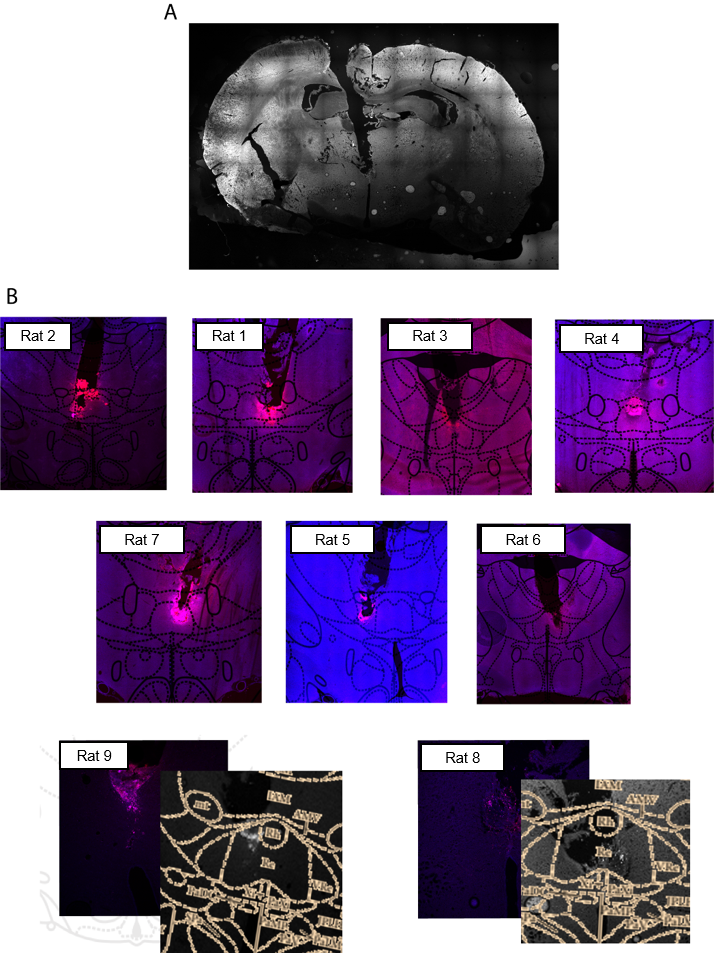


Figure S1. Histological verification of Re inactivation. A) Example brain section. demonstrating cannula placement into the ventral midline thalamus. B) Cannuli and muscimol spread for each rat.


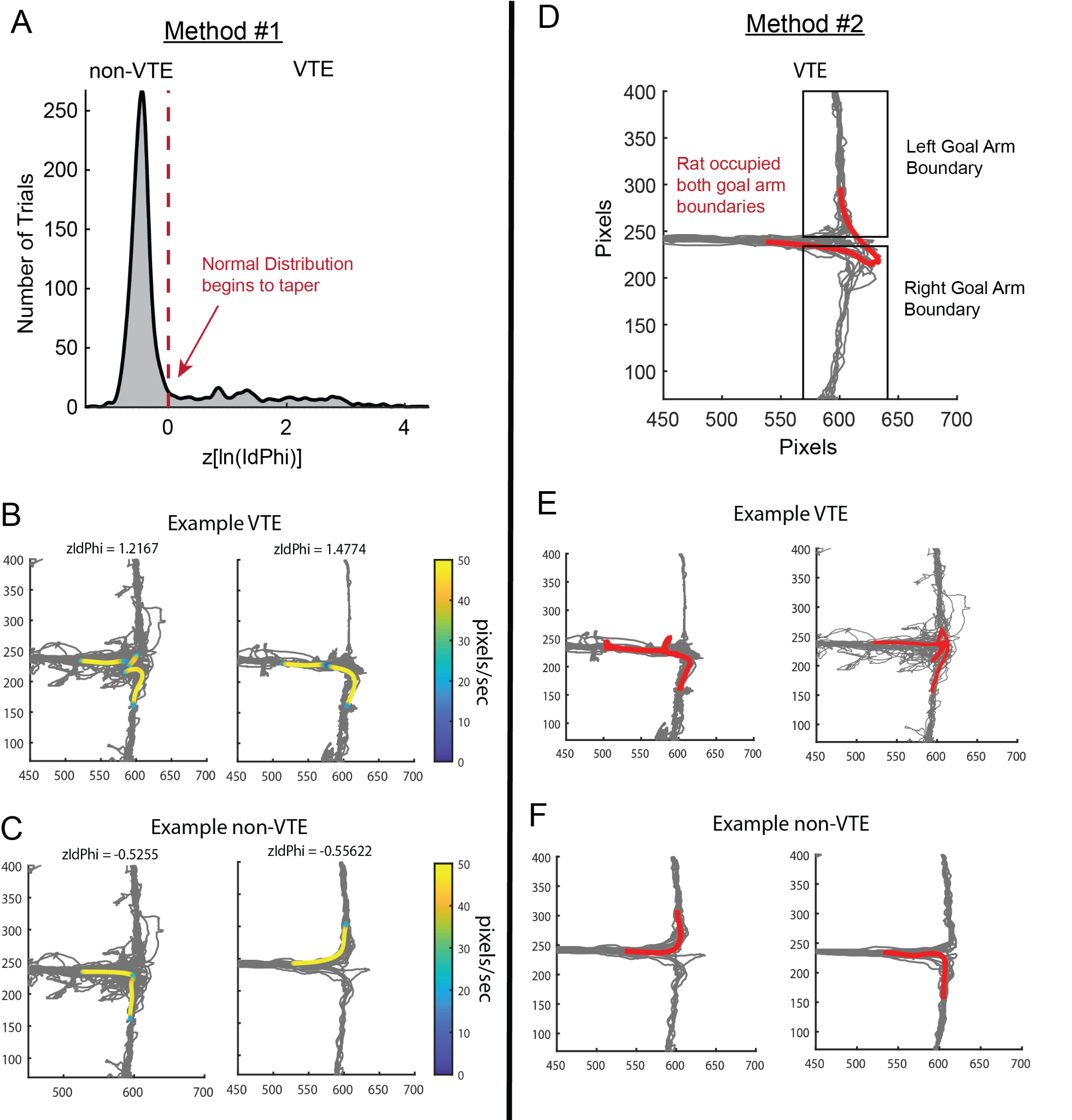


Figure S2. VTE identification. A) Example zlnIdPhi distribution, displayed as the gaussian kernel. The VTE threshold was defined as the first clear deflection of the normal distribution. B) Example VTE and their corresponding zlnIdPhi estimates. C) Example non-VTE and their corresponding zlnIdPhi estimates. D) Head-sweeping VTE’s were further identified by finding trials where rats occupied both goal arms (denoted by black boxes). E) Example VTE. F) Example non-VTE.


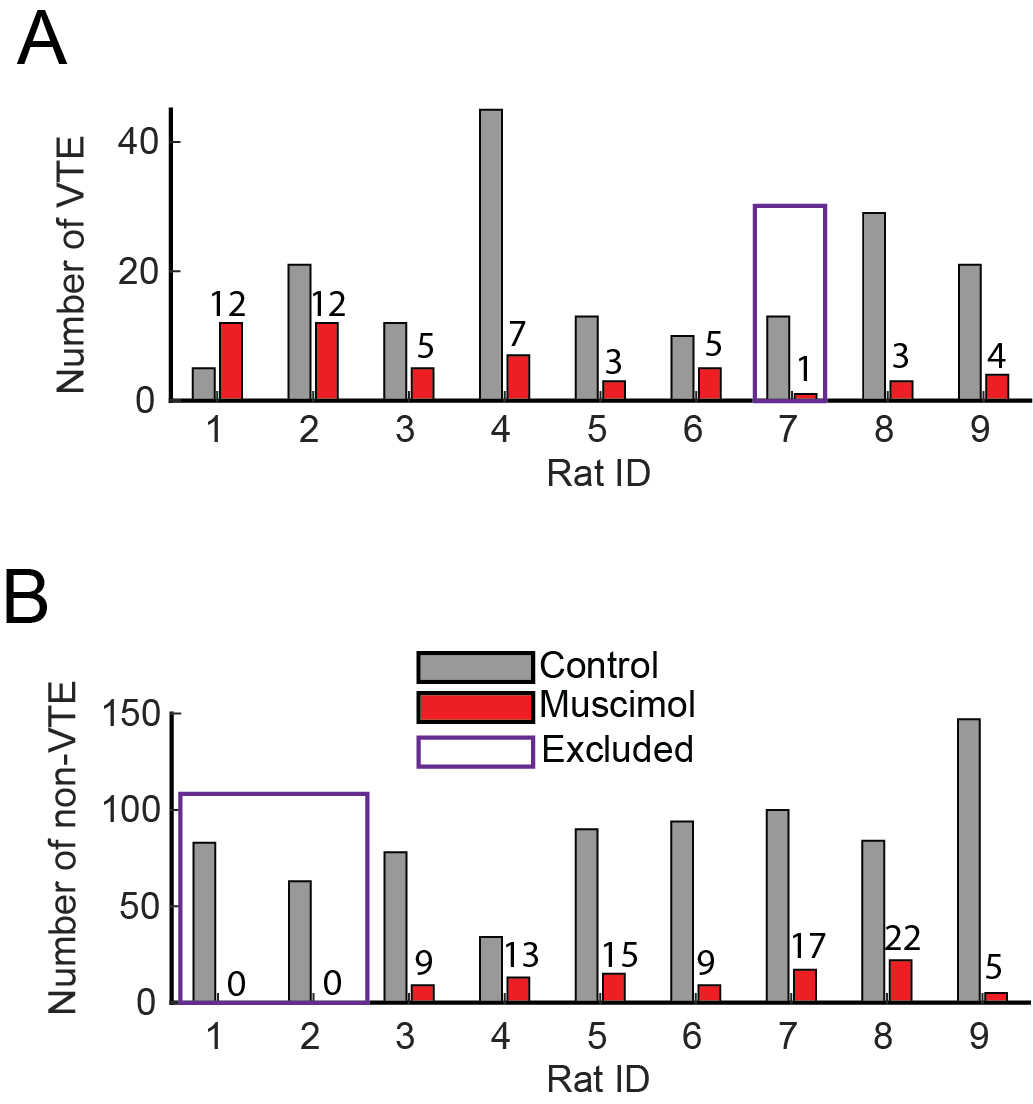


Figure S3. Distributions of VTE and non-VTE trials. A) Distribution of VTE trials among control (gray; all sessions except muscimol testing) and muscimol testing (red). Notice that Rat #7 exhibited 1 VTE and was therefore excluded from all analyses that focused specifically on VTE events. B) Rats #1 and #2 exhibited no non-VTE trials and were therefore excluded from the non-VTE analyses. Notice the sheer difference in trial counts, which required normalization for all analyses.


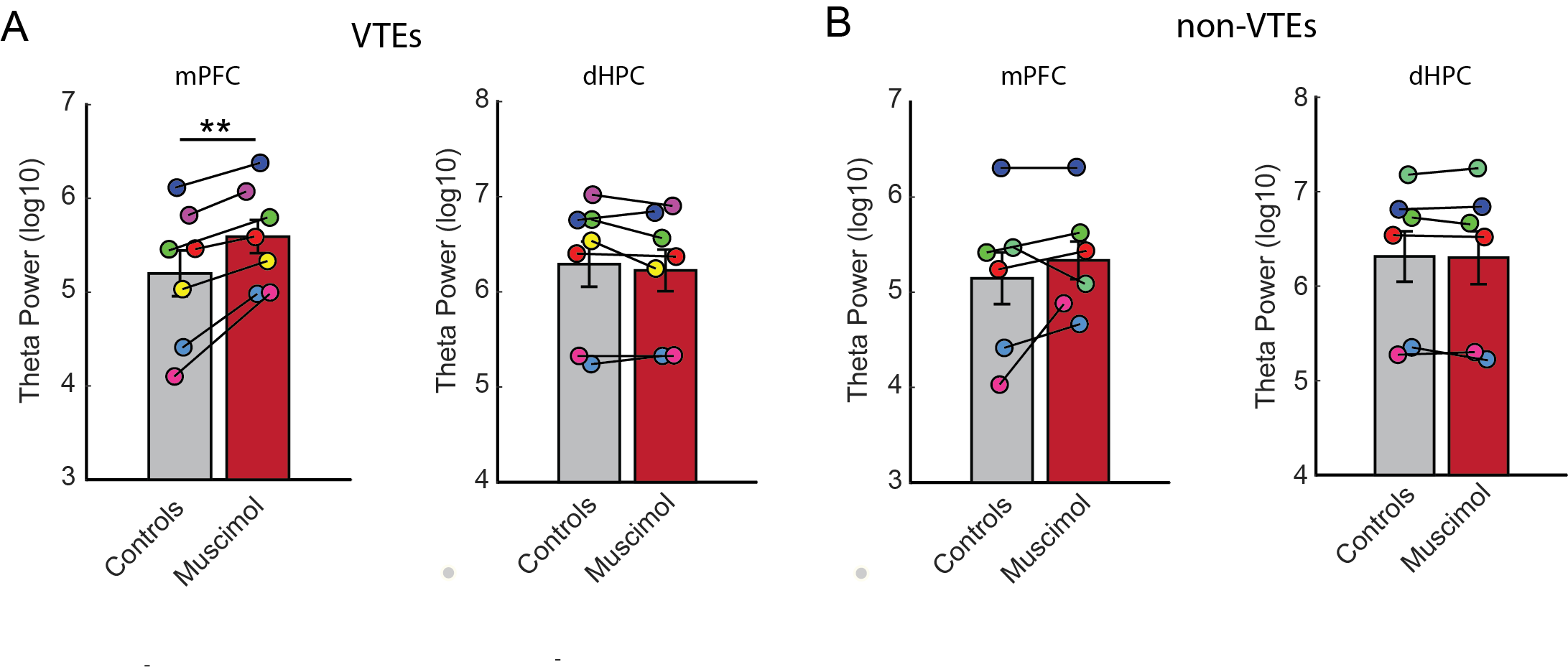


Figure S4. Re suppression led to an increase in mPFC theta power on VTE trials. A) *Left panel:* mPFC Theta (5-10Hz) power on VTE trials was significantly greater under Re inactivation when compared the control sessions. *Right panel:* dHPC theta power did not change under Re inactivation on VTE trials. B) Neither mPFC nor dHPC theta power changed on non-VTE trials when the Re nucleus was inactivated. Data are displayed as the mean ± s.e.m. **p<0.01.
